# Comparative transcriptome analyses of seven anurans reveal functions and adaptations of amphibian skin

**DOI:** 10.1038/srep24069

**Published:** 2016-04-04

**Authors:** Li Huang, Jun Li, Housseni Anboukaria, Zhenhua Luo, Mian Zhao, Hua Wu

**Affiliations:** 1 Institute of Evolution and Ecology, School of Life Sciences, Central China Normal University, 152 Luoyulu, Hongshan District, Wuhan 430079, China

## Abstract

Animal skin, which is the tissue that directly contacts the external surroundings, has evolved diverse functions to adapt to various environments. Amphibians represent the transitional taxon from aquatic to terrestrial life. Exploring the molecular basis of their skin function and adaptation is important to understand the survival and evolutionary mechanisms of vertebrates. However, comprehensive studies on the molecular mechanisms of skin functions in amphibians are scarce. In this study, we sequenced the skin transcriptomes of seven anurans belonging to three families and compared the similarities and differences in expressed genes and proteins. Unigenes and pathways related to basic biological processes and special functions, such as defense, immunity, and respiration, were enriched in functional annotations. A total of 108 antimicrobial peptides were identified. The highly expressed genes were similar in species of the same family but were different among families. Additionally, the positively selected orthologous groups were involved in biosynthesis, metabolism, immunity, and defense processes. This study is the first to generate extensive transcriptome data for the skin of seven anurans and provides unigenes and pathway candidates for further studies on amphibian skin function and adaptation.

The adaptation of organisms to their environments is the basis of evolutionary biology. This adaptation occurs at many levels of biological organization, from molecules to organs^1^. Animal skin, acting as the direct medium interacting with external surroundings, has experienced distinctly adaptive evolution. Numerous shifts have occurred in animal skin functions since the habitats transformed from an aquatic to a terrestrial environment. For aquatic species, the skin mainly possesses basic functions, including protection, sensing, and communication with other organisms, although several specific functions have evolved (*e.g*., locomotion)^2^. Conversely, the skin of species living on land has developed complex functions. Faced with arid environments, terrestrial species have had to adapt their skin to retain moisture. Thus, stratum corneum has developed in their skin, with special structures and compositions^3^,^4^. Moreover, the skin of homeothermic terrestrial animals has developed thermal receptors to deliver signals toward the thermoregulation system^5^. Therefore, the skin of animals has undergone marked changes due to shifts in environmental backgrounds; investigating the differences in skin function among species may shed light on the adaptive evolution of organisms.

Amphibian represents a valuable taxon that links the evolutionary gap between aquatic and terrestrial animals. Their skin performs versatile functions that are important for survival^6^ and possesses not only basic functions, such as acting as the boundary tissue, but also complex structures and adaptive functions. For example, the defensive barriers of amphibian skin are more complex than those of aquatic organisms^2^. The corneous cell envelope, which is an important composition of the amniote stratum corneum, initially occurred in the adult amphibian epidermis^4^. Homologous keratohyalin granules have also increased in the epidermis of amphibians^7^. Furthermore, amphibian skin has developed abundant glands (primarily including mucous and granular glands) that secrete diverse biologically active compounds for protection against predators or microorganisms^8^. The mucous glands usually secrete mucus containing a variety of mucins, which help defend against potential predators^9^. Different from the mucous glands, the granular glands are reservoirs of antimicrobial peptides (AMPs), essential molecules in innate immunity^10^, which provide an effective and fast-acting defense against microorganisms^6^. Additionally, amphibian skin also participates in respiration. In amphibians, at least 30 percent of the oxygen exchange and most or all of the carbon dioxide elimination occurs through the skin^11^. The skin of lungless salamanders (*e.g*., all species in *Plethodontidae* and caecilians) even serves as their only respiratory organ^12^.

Although adaptive functions of amphibian skin have been reported, the underlying molecular basis and functional mechanisms are still unclear. To date, several genes and AMPs have been identified in amphibian skin. For instance, genes of AMP families, such as the *temporin*^13^, *esculetin*^14^ and *brevinin* superfamilies^15^, have been discovered in different taxa. However, most of these researches have used traditional low-throughput methods, such as screening of presumed genes from cDNA libraries, on the basis of conserved regions of precursors. Recently, a high-throughput proteomic analysis on the skin of the Chinese giant salamander has identified several proteins involved in skin functions, including keratins, beta-actins, annexins, and respiration-related proteins^16^; however, this study was restricted to one species and was unable to comprehensively elucidate adaptive molecular evolution. Thus, it is urgent to utilize high-throughput techniques with ample species to explore the molecular basis of amphibian skin functions and adaptation from a holistic perspective.

Emerging transcriptome sequencing technology and bioinformatics tools are undoubtedly the most appropriate methods to address this problem. These tools can obtain the total RNA (including mRNA and noncoding RNA) transcripts from particular cells or tissues^17^. Although many studies have adopted this method to elucidate scientific questions, the existing research on non-model amphibians is insufficient. Most previous studies have focused only on particular development stages or specific tissues^18^,^19^. Species living in different environments evolve diverse skin phenotypes; therefore, comparing the expressed genes in the skin of different amphibians may help us understand the genetic basis of skin functions and adaptation. In this study, we sought to sequence and compare the skin transcripts of different species, to investigate the functions of amphibian skin, and to further reveal the evolutionary significance of this organ. Specifically, we collected seven species widely distributed in the Badagongshan Nature Reserve (Zhangjiajie, Hunan Province). These species belong to three families, *Ranidae*, *Megophryidae* and *Rhacophoridae*, which are semi-aquatic, terrestrial and arboreal, respectively. In the *Ranidae* family, *Pelophylax nigromaculatus* and *Odorrana margaretae* were selected. *Leptobrachium boringii* and *Megophrys sangzhiensis* from the *Megophryidae* family, and *Rhacophorus omeimontis*, *R. dennysi* and *Polypedates megacephalus* from the *Rhacophoridae* family were also chosen. By sequencing and analyzing the transcripts of skin samples from these species, we sought to acquire ample information about the genetic basis of skin functions and adaptation in amphibians.

## Results

### Illumina sequencing and *de novo* assembly

The seven species (four individuals per species) yielded a number of raw reads, ranging from 23,940,046 in *R. omeimontis* to 34,471,587 in *R. dennysi* (Table 1). After removing adapters, primers and low-quality reads, we obtained 19,792,130–33,156,716 clean reads (Table 1). These clean reads were subsequently assembled into transcripts. Finally, the transcripts were assembled into putative unigenes. Detailed information about the putative unigenes in seven species is shown in Table 1. To verify the assembly quality, we mapped the clean reads to the assembled unigenes and BLASTed the available protein sequences of a closely related species (*Xenopus tropicalis*) with assembled unigenes. The ranges of mapping rates and BLAST rates in seven species were 72.99–84.01% and 50.02–56.94%, respectively. The raw reads were available in the NCBI SRA browser (Bioproject accession number: SRR2768939), and the sequences of assemblies were deposited in the Transcriptome Shotgun Assembly (TSA) Database under accession numbers: GEGF00000000 for *R. omeimontis*, GEGG00000000 for *R. dennysi*, GEGH00000000 for *P. megacephalus*, GEGI00000000 for *P. nigromaculatus*, GEGJ00000000 for *O. margaretae*, GEGK00000000 for *L. boringii* and GEGL00000000 for *M. sangzhiensis*.

### Functional annotation of the unigenes

The entire set of unigenes was annotated in seven frequently used databases, including the NCBI non-redundant (Nr) protein and nucleotide (Nt) databases, the Protein family (Pfam) database, the Cluster of Orthologous Groups of proteins (KOG/COG) database, the SwissProt protein database, the Kyoto Encyclopedia of Genes and Genomes (KEGG) Ortholog (KO) database, and the Gene Ontology (GO) database. Among the total unigenes, approximately 42.99% to 50.38% had been annotated in at least one database according to BLAST searches (Table 2).

**Figure 1:**
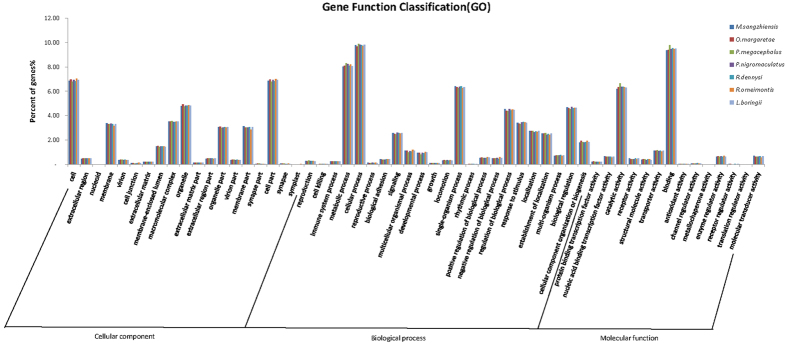
Histogram of the GO annotations of unigenes in seven species. The *x*-axis represents GO terms belonging to three categories, and the *y*-axis represents gene percentages of each term. Bars with different colors represent different species.

To identify the functional distribution of unigenes, we collected GO annotations for seven species. The annotated unigenes ranged from 18,776 in *M. sangzhiensis* to 24,555 in *P. megacephalus* (Table 2). Intriguingly, the distribution of annotated unigenes at level two GO terms in the seven species showed highly similar patterns (Fig. 1). All of the unigenes annotated in the GO database were classified into three main categories (Fig. 1). Among them, “biological processes” contained the largest numbers of annotated unigenes, followed by “cellular components” and “molecular functions”. Within the biological processes, “cellular process” and “metabolic process” were the two most common GO terms. For the cellular components category, most of the unigenes were related to “cell part” and “cell”. In the category of molecular functions, unigenes were mostly involved in “binding”, followed by “catalytic activity” (Fig. 1). These highly enriched GO terms mainly referred to the maintenance of the basic regulation and metabolic functions of amphibian skin. Additionally, GO terms related to special skin functions, such as “response to stimulus” in the biological process category and “oxidoreductase activity” in the molecular function category, were also enriched. To further annotate the gene functions, we analyzed the GO annotations at level three. “Oxidoreductase activity” accounted for 16.9–18.6% of unigenes in the seven anurans.

**Figure 2:**
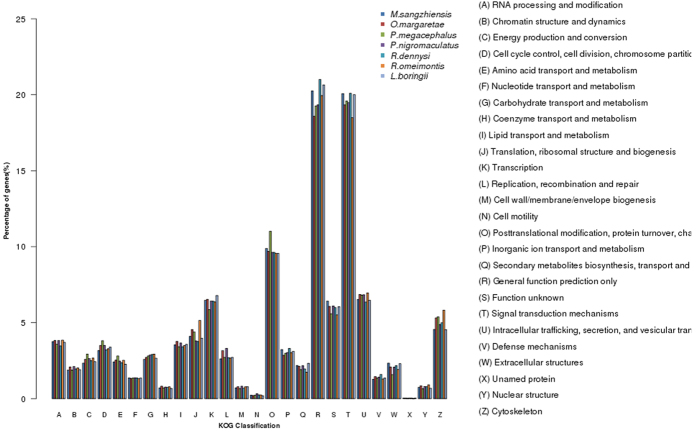
KOG annotation results of the skin of seven anuran species. The capital letters on the *x*-axis symbolize 26 biological processes, as shown on the right side, and the *y*-axis represents the gene percentage of each process. Bars with different colors represent different species.

In addition to the GO annotation, we mapped the unigenes in the KOG categories and found similar distribution patterns across the seven anurans. The percentage of annotated unigenes in the “general function prediction only” (“R” term) and “signal transduction mechanisms” (“T” term) categories were far higher than those in the other categories, containing more than 15% of the unigenes (Fig. 2). Moreover, several terms relating to skin-specific functions, such as intracellular trafficking, secretion, and vesicular transport (U) and defense mechanisms (V), were also enriched (Fig. 2).

**Figure 3:**
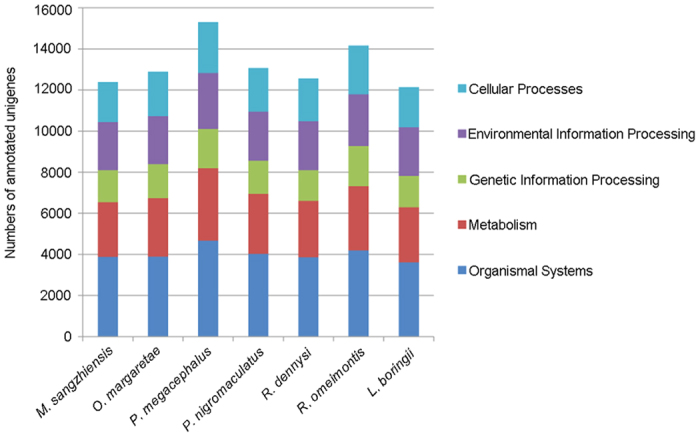
KEGG pathway annotation for the skin of seven anuran species. Bars with different colors represent five pathways in KEGG annotations. The *x*-axis represents the seven species, and the *y*-axis represents the total annotated transcript numbers.

Additionally, we performed KEGG pathway analysis to understand the biological functions and interactions among gene products. Unigenes annotated in the Nr and Pfam databases were searched in the KEGG database. Five types of hierarchy one pathways were involved in the KEGG annotation in the seven species (Fig. 3). Among them, the “organismal systems” pathway, including the categories “circulatory system”, “development”, “digestive system”, “endocrine system”, “environmental adaptation”, “excretory system”, “immune system”, “nervous system”, and “sensory system”, contained the largest number of annotated sequences. In hierarchy two pathways, most of the unigenes were annotated in signal transduction pathways across seven species. Notably, the PI3K-Akt signaling pathway (ko: 04151, Supplementary Fig. S1) was the top enriched pathway in all seven species.

### The immunome and antimicrobial peptides (AMPs) of seven anurans

For amphibian skin, immune functions, especially AMP secretion, are among the most important functions. KEGG pathway analysis also revealed the highly enriched immune-related pathways in seven species. Therefore, we sought to explore the molecular basis of skin immune function in amphibians. First, the unigenes in immune system processes (GO annotation) were counted in the seven species (Table 3). Then, the differences in numbers of unigenes annotated in each process were evaluated among three families by using the analysis of variance (ANOVA). Among the 16 processes of the immune system (level two GO categories), “immune system development”, “regulation of immune system process” and “immune response” contained the three largest amounts of unigenes (Table 3), indicating their important roles in the immune system. For the differences among the three families, the numbers of unigenes annotated in “activation of immune response” (level two category) and “immune response-activating signal transduction” (level three category) differed significantly among the three families, with ANOVA *F* = 8.399, *P* = 0.037 and ANOVA *F* = 10.156, *P* = 0.027, respectively. Second, all unigenes were mapped in the database of anuran defense peptides (DADP) to identify AMPs across seven species. In total, 108 putative AMPs were identified in these skin samples. The numbers of AMP unigenes identified in single species varied from 4 in *L. boringii* to 28 in *O. margaretae* (Table 4). Notably, the midkine family was present in the skin of all anurans, thus indicating its potential role in defense against microorganisms. The overall types of AMPs showed differences among the three families, but these differences were marginally non-significant (ANOVA *F* = 6.094, *P* = 0.061), and species in the *Ranidae* family produced more AMPs than did species in the other families.

### Quantification of gene expression in seven anurans

To quantify the expressed levels of unigenes, we separately calculated the FPKM (fragments per kb per million reads) values and ranked the values from high to low in seven species (Supplementary Dataset 1). For *O. margaretae* and *P. nigromaculatus*, which belong to the *Ranidae* family, AMP unigenes (such as esculentin-2-MG1, esculentin-1-MG1, and pelophylaxin-2 protein precursor) were expressed with extremely high FPKM values (Supplementary Dataset 1). However, similar results were not found for species in the other two families (Supplementary Dataset 1). Furthermore, unigenes of keratin-associated proteins (type I and II) were extremely highly expressed in the skin of the *Rhacophoridae* and *Megophryidae* families, but the expression levels of the same unigenes were relatively low in species of the *Ranidae* family. Additionally, the oxidative cytochrome *c* oxidase 1 (COX1) unigenes were most highly expressed in the skin of only the *Megophryidae* family, and ferritin genes were most highly expressed in the skin of species from both the *Rhacophoridae* and *Ranidae* families. Next, we searched these special unigenes in the GO annotation results and found that they mainly involved “metabolic process”, “binding”, “response to stimulus”, “catalytic activity”, and “enzyme regulator activity”. The AMPs were involved in “defense response” (level four), “response to stress” (level three), and “response to bacterium” (level three), whereas keratins were involved in the level four GO term “response to wounding” and the level three GO terms “response to stress” and “cytoskeletal protein binding”. Both COX1 and ferritin were involved in “oxidoreductase activity”. The most highly expressed genes were related to the respiration (“catalytic activity”), protection (“response to wounding”) and immunity (“response to stimulus”) functions of amphibian skin.

### Selection detection

A total of 3,280 orthologous groups (OGs) were identified across seven species. Then, a phylogenetic tree of these OGs was constructed to detect selective genes (Supplementary Fig. S2). Seven OGs were detected under strong positive selection, with false discovery rate (FDR) corrected *q* values lower than 0.05 (Table 5). Subsequently, we annotated the protein functions of these OGs against the Nr database and found that these OGs were predicted to be TCEA [transcription elongation factor A (SII)], ATPIF1 (ATPase inhibitory factor 1), EIF3C (eukaryotic translation initiation factor 3 subunit C), ANXA1 (annexin A1), CAPZB (F-actin capping protein subunit beta 2), CD9 (CD9 antigen) and putative RPL26L1 (ribosomal protein l26-like 1). Furthermore, we searched these seven OGs against the KEGG database to annotate the pathways in which they are involved. Only three pathways were predicted: ribosomes (ko: 03010), hematopoietic cell lineage (ko: 04640), and RNA transport (ko: 03013). Among them, the hematopoietic cell lineage pathway, in which the CD9 antigen gene is predicted to function (Supplementary Fig. S3), is involved in the KEGG (hierarchy two) immune system pathway.

## Discussion

Exploring the molecular basis of skin function and adaptation in amphibians is essential to understand the survival and evolutionary mechanisms of amphibians and amniotes. In this study, we generated extensive transcriptome datasets of the skin in seven anurans, compared the unigene populations and expression patterns, and finally inferred the candidate genes involved in skin adaptive evolution. By sampling the skin of four individuals per species and sequencing their transcriptomes, we acquired more than 20 million reads for each species and over 210 million reads for the skin of seven species. This large dataset allowed us to examine the skin function of amphibians in detail and to identify novel genes and proteins involved in skin adaptation.

According to the functional annotation results, unigenes that participated in skin’s basic biological and metabolic processes were largely enriched. In the level two GO categories of cellular component and biological process, most of the unigenes were involved in maintaining basic biological functions, such as “cell part” and “metabolic process”, indicating the basic functions of amphibian skin. In molecular function terms, many of the identified unigenes participate in “binding” processes, including ion binding, heterocyclic compound binding, and organic cyclic compound binding, thus implying that amphibian skin is active in synthesizing the proteins involved in these processes. Similar findings have been reported in Chinese brown frogs^20^. Furthermore, unigenes of the ribosomal proteins were highly expressed in the skin of all seven frogs. Ribosome proteins (40S and 60S) are similarly abundantly expressed in the skin of channel catfish^21^ and sheep^22^. The abundant expression of these ribosomal protein genes is presumably due to increased peptide synthesis.

In addition to its basic survival functions, amphibian skin has evolved complex structures and functions that allow amphibians to live between water and land^2^. The skin acts as the first-line defensive barrier against harmful external factors. In this study, two types of keratins (type I and II) were identified on the basis of their high expression levels in species of the *Megophryidae* and *Rhacophoridae* families, thus indicating their important biological functions. In GO annotation, keratin unigenes were annotated as “response to stress” and “response to wounding processes”. In addition, previous studies have recorded that keratins are the most versatile, plentiful constituents involved in cell binding, adhesion, and signal transport functions^23^. These proteins may also play general roles in skin integrity and disease defense^24^. Therefore, we presumed that the keratins in amphibian skin play an essential role in protection, and further studies on these types of genes are needed.

Furthermore, amphibian skin has developed functions in immunity to defend against various external microbes. As the annotation results showed, the abundant processes related to “response to stimulus” of the biological process term in the GO classification, “defense mechanisms (V)” in the KOG annotation, and “immune system” of the organismal systems term in the KEGG pathways demonstrated the functions of immunity and defense in anuran skin, although the expression levels of these genes were not the highest among the studied genes. This phenomenon is plausible because the immune system involves many types of tissues, such as the thymus, spleen and skin, and the number of contigs annotated for the immune system function in the skin occupies a relatively small proportion (28.48%) of the total contigs in immune tissues^19^. In addition, the immune-related PI3K-Akt signaling pathway, which plays critical roles in immune cell development^25^, was the top enriched pathway in the seven anurans. Therefore, we presumed that immune cell development is a common process in amphibian skin. The skin’s immune functions are executed mostly by AMPs, which are secreted and released by skin granular glands and protect organisms against harmful pathogens^6^. Because of their important role in immunity, AMPs have been characterized across diverse amphibians through traditional low-throughput methods^6^,^13^,^14^,^15^. However, these methods are limited and are not comprehensive, owing to the presumably targeted AMP genes. In this study, a high-throughput transcriptome sequencing method was used to identify as many AMPs as possible at one time. Overall, we identified 108 AMP unigenes belonging to 19 families. Among these unigenes, only the MDK (midkine) family was identified in all the seven species. MDK was first characterized in murine carcinoma cells, and it plays important roles in the release of growth factors during inflammation^26^. Its ortholog was also present in *X. laevis*^27^ but was not present in other amphibians. Thus, this study provided evidence that MDK exists in the skin of these seven non-model species; further research on its potential functions is needed.

Respiration is another important function of amphibian skin^11^,^12^. As shown in this study, the process of “catalytic activity” was detected in the seven frog skin samples according to the GO annotation at level two. In this activity, oxidoreductases, such as mitochondrial complex I: NADH-ubiquinone oxidoreductase, have been represented as an important enzyme type in the respiratory chain^28^. Moreover, three types of oxidoreductase activity genes [COX1, COX3 (cytochrome *c* oxidase 3) and GAPDH (glyceraldehydes-3-phosphate dehydrogenase)] possess relatively high FPKM values in *Ranidae* and *Megophryidae* species. It has been reported that the mitochondrial COX proteins are important electron-driven proton pumps and participate in cell respiration and aerobic energy metabolism^29^. In addition, recent studies on the proteome of the Chinese giant salamander have identified several proteins involved in respiration, such as NADH-ubiquinone oxidoreductase chain 5, cytochrome b and ATPase subunit 6^16^. Therefore, we suggest that future research on the relationships among these genes is necessary to elucidate the molecular mechanism of skin respiration in amphibians.

To deduce the adaptability of skin, we compared the similarities and differences in the unigene expression levels among species from three families. The types of highly expressed genes were different but showed similar functions among the three families. Cystatins (CSTs) and AMPs, which were, highly expressed in species of Rhacophoridae and Ranidae, respectively, are all related to immune functions. An earlier report has noted that CSTs, including cystatin and stefin, have important physiological functions in the immune system, including stabilizing host-parasite interactions and defending against pathogens^30^. Similarly, both COX genes and ferritin subunits, which were highly expressed in species of Megophryidae and the other two families, respectively, are involved in the GO term “oxidoreductase activity”, indicating their similar roles in respiration. Therefore, all species exhibited highly expressed genes in defense, immune functions, and respiration, but each exhibited different types of genes and proteins. It has been noted that frogs’ phenotypes are correlated with their ecological habitats^31^; thus, we hypothesized that these discrepancies among families may be linked to their diverse habitats. *Megophryidae*, *Rhacophoridae* and *Ranidae* species live in terrestrial, arboreal, and semi-aquatic habitats, respectively. Semi-aquatic species are exposed to more pathogens than terrestrial and arboreal species because water often serves as a transmission medium for pathogens such as the zoospore of *Batrachochytrium dendrobatidis*^32^. Hence, AMPs were highly expressed in the skin of only the *Ranidae* family species (*O. margaretae* and *P. nigromaculatus*), and the AMP categories also suggested that the *Ranidae* family expressed and produced more antimicrobial proteins than the other two families did. Nevertheless, this finding does not mean that terrestrial and arboreal species lack immune defense capacity; instead, they have evolved with different protection mechanisms to adapt to complex environments. As shown in the FPKM ranking of seven species, keratins, which are associated with maintaining cell binding and adhesion^23^, were all highly expressed in *Megophryidae* and *Rhacophoridae* species. Tight cytoskeletal structures protect organisms against microorganisms and help heal wounds. The high expression of keratin genes in *Megophryidae* and *Rhacophoridae* species indicates that these species possess strong skin defense functions that differ from those of *Ranidae* species. Furthermore, for semi-aquatic species of *Ranidae*, two types of genes related to respiration were highly expressed, thus suggesting that these species require these highly expressed genes to ensure skin respiration, especially when they reside in water with low oxygen concentrations. Thus, these different patterns of highly expressed genes among families may tightly correlate with their habitats.

During evolution, positive selection, which suggests an excess of high-frequency variants leading to changes in encoded proteins, is a frequent event for the formation of new genes and functions^33^. Recently, high-throughput transcriptome sequencing has offered a new technique for discovering positively selected genes; many genes have been identified through this method^33^,^34^. In this study, seven OGs exhibited strong positive selection, with functions involving biosynthesis, metabolism, immunity and defense processes. Specifically, TCEA, EIF3C and RPL26 all play important roles in translation and protein synthesis^35^,^36^,^37^, whereas ATPIF1 is an essential element regulating ATP synthesis, hydrolysis, and energy metabolism^38^. In immunity and defense processes, three proteins were identified to be positively selected: CAPZB, ANXA1 and CD9. CAP (F-actin capping protein) is an actin-binding protein that regulates the function of the actin system and maintains cytoskeletal organization^39^. This protein affects the length and number of actin filaments via binding to the barbed end of actin filaments^39^. Therefore, CAP is involved in skin structure maintenance and stable wound healing. ANXA1 might effectively mediate the anti-inflammatory activity of glucocorticoids, thereby affecting the innate and adaptive immune systems^40^. These two proteins are both involved in maintaining the defense barrier functions of skin. CD9 was originally described as a surface marker of leukemia and lymphohematopoietic cells; it participates in T cell activation, which serves as the initiation process of the immune response^41^. A recent study has indicated that this marker may be involved in the anti-bacterial immune response in turtles^42^. KEGG pathway annotation also indicated that CD9 participates in the hematopoietic cell pathway of the immune system (as shown in Supplementary Fig. S3). Therefore, it is likely that these positively selected genes, which are involved in biosynthesis, metabolism, immunity, and defense processes, are targets of adaptive molecular evolution. New traits resulting from changes in these proteins may allow species to adapt to their habitats^34^.

In summary, we sequenced the skin transcriptomes of seven species belonging to three families to explore the molecular mechanisms and adaptive evolution of amphibian skin. Many genes and proteins involved in basic and specific skin functions were identified. The highly expressed genes in different species were related to their corresponding habitats. Species living in different environments may evolve diverse adaptive mechanisms with different molecular bases. Seven OGs involved in biosynthesis, metabolism, immunity, and defense processes were presumed to be candidates of adaptive molecular evolution. This study provides molecular data for exploring the mechanisms of skin function in amphibians and may serve as a guide for researchers studying the adaptation of skin at the molecular level.

## Methods

### Ethics statement

All samples used in this study were collected with the permission of the management bureau of the Badagongshan National Nature Reserve. The animal experiments were performed under an animal ethics approval granted by the Central China Normal University (CCNU).

### Sample collection and RNA extraction

A total of 28 frogs belonging to the three families were collected from Badagongshan National Nature Reserve (with permission) in Hunan province during the 2013 breeding season. They were separately placed in single plastic boxes (173 × 115 × 68 mm) containing the same plant leaves and water as their natural environment, and they were brought back to the laboratory. The living frogs were then sacrificed and dissected with scissors soaked in a 0.1% diethyl pyrocarbonate (DEPC) solution in the laboratory. For each species, the skin tissue was carefully removed from the underlying tissues and flash-frozen in liquid nitrogen until needed. These experiments were performed in accordance with relevant animal ethics guidelines and regulations granted by CCNU. To eliminate the individual differences and to identify as many expressed genes as possible, we mixed skin tissues from four individuals (two female and two male) per species and extracted the total RNA using an RNA extraction kit (Omega Bio-Tek, USA) together with TRIzol (Invitrogen, USA). RNA degradation and contamination was assessed with 1% agarose gels. The purity, on the basis of optical densities of the samples, was examined using a NanoPhotometer^®^ spectrophotometer (Implen, USA). RNA concentration was measured with a Qubit^®^ RNA Assay Kit with a Qubit^®^ 2.0 Fluorometer (Life Technologies, USA). The RNA integrity was assessed using an RNA Nano 6000 Assay Kit with a Bioanalyzer 2100 system (Agilent Technologies, USA). Additionally, to measure the RNA quality of each sample, the RNA Integrity Number (RIN) was calculated^43^. All seven samples had RIN values larger than 7.8 (7.8–9.4), indicating the high quality of the input RNA samples.

### cDNA library construction and sequencing

A total amount of 3 μg of RNA per species was used to construct sequencing libraries. The mRNAs were enriched with oligo - (dT) beads and were then broken into short fragments with NEBNext First Strand Synthesis Reaction Buffer (5×). These fragments were subsequently used as templates to synthesize the first-strand and second-strand cDNAs. After adenylating the 3′ ends of the cDNA fragments, NEBNext Adaptors were ligated to the cDNA fragments. The cDNA fragments of 150–200 bp were preferentially purified with an AMPure XP system (Beckman Coulter, USA). Subsequently, the purified adaptor-ligated cDNAs were successively treated with USER Enzyme (NEB, USA) at 37 °C for 15 min and 95 °C for 5 min. Then, PCR was conducted using Phusion High-Fidelity DNA polymerase, universal PCR primers and an Index (X) primer. PCR products were purified using an AMPure XP system, and the library quality was evaluated using an Agilent Bioanalyzer 2100 system. Subsequently, the cDNA samples were clustered on a cBot Cluster Generation System using a TruSeq PE Cluster Kit (Illumina, USA). After cluster generation, the library preparations were sequenced on a HiSeq^TM^ 2000 platform (Illumina, USA), and the stranded paired-end reads were generated.

### Sequence assembly and annotation

All of the raw data were initially processed using in-house Perl scripts (Supplementary Methods). Prior to assembly, clean reads were obtained by removing the sequencing adapters, primers, and low-quality reads (reads including bases greater than 50% with a *Q*-value ≤ 5 and ambiguous bases (N) more than 10%). The clean reads were assembled *de novo* in Trinity (r2012-10-05)^44^ with 2 for the min_kmer_cov setting and default settings for all other parameters. The unigenes were identified as the longest transcript for each gene with in-house Perl scripts to avoid redundant transcripts (Supplementary Methods). Unigenes generated by assembly were subsequently used for downstream analyses.

All assembled unigenes were annotated using NCBI-BLAST-2.2.28 + with *E*-values ≤ 1E-5 in seven databases, including the Nr and Nt databases in NCBI (http://www.ncbi.nlm.nih.gov), the Pfam database (http://en.wikipedia.org/wiki/Protein_family), the KOG (http://www.ncbi.nlm.nih.gov/COG), the SwissProt protein database (http://www.expasy.ch/sprot), the KO database (http://www.genome.jp/kegg/pathway.html), and the GO database (http://www.geneontology.org/). To assess the quality of assembled reads, we mapped the total clean reads of seven species to the assembled unigenes using the Bowtie mode of RSEM 1.2.0^45^,^46^. Furthermore, we BLASTed the available protein sequences of *X. tropicalis* (ftp://ftp.ensembl.org/pub/release-75/fasta/xenopus_tropicalis/) with our assembled unigenes using NCBI-BLAST-2.2.28 + with *E*-values ≤ 1E-5. Additionally, we performed GO annotation^47^ to classify the functions of our unigenes utilizing the Blast2GO pipelines^48^ with *E*-values ≤ 1E-3, retaining the top 20 hits. Then, we searched against the eukaryotic KOG database^49^ to annotate the unigenes with *E*-values ≤ 1E-5. Additionally, we searched sequences against the KEGG database to understand the functions and pathway annotations using KOBAS 2.0^50^.

### AMP identification

The AMPs were identified by scanning the database of anuran defense peptides (DADP)^51^ using NCBI-BLAST-2.2.28 + with *E*-values ≤ 1E-5. Additionally, AMPs that were not found in the DADP database but that have been described in the literature for amphibians, such as the cathelicidin family^52^, were set as keywords when searching against the Nr database. Furthermore, the keyword “andersonin-9” (GenBank: GU134093.1), which is a type of AMP found in *Odorrana andersonii* and our species but was absent in the DADP database, was also included. The types of AMPs were determined by the Nr annotation and DADP scanning results. Then, we assessed the significant differences in the categories of AMPs of frogs in the three families using one-way ANOVA analysis.

### Quantification of gene expression levels

We used the software package RSEM 1.2.0 to estimate the gene expression levels for each species. First, read_count values of all genes were obtained by mapping clean data back onto the transcripts. Then, we calculated the FPKM values, the most common method to estimate gene expression levels^53^. After estimating the FPKM values, we searched the Nr database to predict proteins and domains and then annotated the biological functions using GO classifications. The FPKM values were ranked from high to low for each species.

### Selection analyses

To recognize the molecular basis of adaptive selection, we conducted selection detection across all orthologous genes. OrthoMCL 2.0.3^54^, which is based on protein similarity graph methods, was used to identify putative OGs from assembled sequences among the seven species. To identify positively selected genes, we reconstructed the orthologous genes tree using maximum likelihood (ML) methods in PhyML 3.0^55^. The outgroups were *X. tropicalis* and *Latimeria chalumnae. Site models* in the “Codeml” application of PAML 4.7^56^ were then used to detect the positively selected genes among putative OGs of the seven species. Generally, *q* values that were corrected by FDRs lower than 0.05 indicated significant evidence of positive selection.

## Additional Information

**How to cite this article**: Huang, L. *et al*. Comparative transcriptome analyses of seven anurans reveal functions and adaptations of amphibian skin. *Sci. Rep*. **6**, 24069; doi: 10.1038/srep24069 (2016).

## Supplementary Material

Supplementary Information

Supplementary Table S1

## Figures and Tables

**Table 1:** Statistical summary of the assembled unigenes in the skin of seven frogs.

Species	Raw reads	Clean reads	Total numbers	Min Length	Mean Length	Max Length	N50	BLAST rates	Mapping rates
*M. sangzhiensis*	33,897,689	32,703,903	48,794	201	868	15,756	1,652	55.32%	84.01%
*L. boringii*	30,103,264	29,086,874	49,267	201	840	18,108	1,578	56.94%	80.23%
*P. megacephalus*	31,428,218	30,529,842	69,425	201	699	17,281	1,142	51.29%	80.42%
*R. dennysi*	34,471,587	33,156,716	63,614	201	770	17,309	1,387	54.78%	82.26%
*R. omeimontis*	23,940,046	19,792,130	55,841	201	626	15,364	893	50.02%	72.99%
*O. margaretae*	24,211,333	19,888,107	54,093	201	687	15,166	1,046	51.82%	81.41%
*P. nigromaculatus*	32,146,280	31,173,113	61,691	201	690	15,507	1,127	54.80%	78.71%

Note: BLAST rates were generated by BLASTing the closely related *X. tropicalis* protein databases with the assembled unigenes. Mapping rates were generated by mapping clean reads to the assembled unigenes using the Bowtie mode of RSEM 1.2.0.

**Table 2:** Numbers of unigenes annotated in the skin of the seven anuran species against seven databases.

	Megophryidae		Rhacophoridae			Ranidae	
	*M. sangzhiensis*	*L. boringii*	*P. megacephalus*	*R. dennysi*	*R. omeimontis*	*O. margaretae*	*P. nigromaculatus*
NR	21,500(44.06%)	21,728(44.1%)	27,028(38.93%)	23,269(36.57%)	24,514(43.89%)	22,532(41.65%)	23,069(37.39%)
NT	10,875(22.28%)	10,935(22.19%)	11,609(16.72%)	11,270(17.71%)	11,226(20.1%)	11,055(20.43%)	11,528(18.68%)
KO	11,318(23.19%)	11,237(22.8%)	13,742(19.79%)	11,847(18.62%)	12,912(23.12%)	11,865(21.93%)	11,829(19.17%)
SP	19,873(40.72%)	19,924(40.44%)	23,936(34.47%)	21,008(33.02%)	22,424(40.15%)	20,670(38.21%)	20,989(34.02%)
Pfam	16,476(33.76%)	16,590(33.67%)	21,712(31.27%)	18,438(28.98%)	17,171(30.74%)	16,747(30.95%)	17,432(28.25%)
GO	18,776(38.48%)	18,874(38.3%)	24,555(35.36%)	20,818(32.72%)	20,510(36.72%)	19,540(36.12%)	20,209(32.75%)
KOG	11,083(22.71%)	11,008(22.34%)	13,810(19.89%)	11,356(17.85%)	11,536(20.65%)	10,970(20.27%)	11,000(17.83%)
one	24,583(50.38%)	24,749(50.23%)	32,176(46.34%)	27,352(42.99%)	27,657(49.52%)	25,908(47.89%)	27,182(44.06%)
Total	48,794(100%)	49,267(100%)	69,425(100%)	63,614(100%)	55,841(100%)	54,093(100%)	61,691(100%)

The percentages of unigenes annotated against each database among all unigenes are shown in parentheses.

Note: SP, SwissProt; one indicates unigenes annotated in at least one database.

**Table 3:** Numbers of unigenes annotated for immune functions and their percentages in all processes in the seven anuran species.

Immune system process	Megophryidae		Rhacophoridae			Ranidae	
	*M. sangzhiensis*	*L. boringii*	*P. megacephalus*	*R. dennysi*	*R. omeimontis*	*O. margaretae*	*P. nigromaculatus*
Immune system development	90(52.02%)	81(56.64%)	95(52.49%)	90(56.60%)	101(56.42%)	89(57.42%)	88(62.86%)
Regulation of immune system process	84(48.55%)	79(55.24%)	91(50.28%)	80(50.31%)	94(52.51%)	77(49.68%)	73(52.14%)
Immune response	69(39.88%)	55(38.46%)	81(44.75%)	63(39.62%)	67(37.43%)	55(35.48%)	52(37.12%)
Positive regulation of immune system process	55(31.79%)	43(30.07%)	54(29.83%)	48(30.19%)	46(25.70%)	39(25.16%)	43(30.71%)
Leukocyte activation	53(30.64%)	33(23.08%)	59(32.60%)	55(34.59%)	53(29.61%)	38(24.52%)	43(30.71%)
Immune effector process	36(20.81%)	23(16.08%)	40(22.10%)	30(18.87%)	35(19.55%)	28(18.06%)	26(18.57%)
Activation of immune response	31(17.92%)	24(16.78%)	28(15.47%)	22(13.84%)	24(13.41%)	20(12.90%)	20(14.29%)
Myeloid cell homeostasis	19(10.98%)	19(13.29%)	20(11.05%)	20(12.58%)	25(13.97%)	21(13.55%)	24(17.14%)
Negative regulation of immune system process	13(7.51%)	18(12.59%)	12(6.63%)	12(7.55%)	12(6.70%)	12(7.75%)	18(12.86%)
Leukocyte migration	14(8.09%)	11(7.69%)	11(6.08%)	11(6.92%)	13(7.27%)	11(7.10%)	14(10.00%)
Production of molecular mediator of immune response	11(6.36%)	8(5.59%)	10(5.52%)	8(5.03%)	10(5.59%)	9(5.81%)	10(7.14%)
Antigen processing and presentation	10(5.78%)	3(2.10%)	7(3.87%)	6(3.77%)	5(2.79%)	6(3.87%)	5(3.57%)
Somatic diversification of immune receptors	7(4.05%)	3(2.10%)	7(3.87%)	5(3.14%)	9(5.03%)	6(3.87%)	8(5.71%)
Leukocyte homeostasis	6(3.47%)	3(2.10%)	3(1.66%)	3(1.89%)	4(2.23%)	6(3.87%)	3(2.14%)
T cell selection	4(2.31%)	2(1.40%)	3(1.65%)	3(1.89%)	0	1(0.65%)	1(0.71%)
Tolerance induction	1(0.58%)	0	1(0.55%)	1(0.63%)	3(1.68%)	2(1.29%)	0
All	173	143	181	159	179	155	140

**Table 4:** Numbers of AMP transcripts in the seven anuran species.

AMP families	Megophryidae		Rhacophoridae			Ranidae	
	*M. sangzhiensis*	*L. boringii*	*P. megacephalus*	*R. dennysi*	*R. omeimontis*	*O. margaretae*	*P. nigromaculatus*
Antimicrobial peptide precursor						1(1)	
Andersonin			4(1)	8(1)	2(1)	4(1)	
Bombesin	1(1)		1(1)			1(1)	
Brevinin	1(1)					1(1)	1(1)
Bradykinin							2(2)
Cathelicidin	2(1)		1(1)	3(2)	4(3)	3(3)	4(4)
Esculentin	1(1)		1(1)	2(2)	1(1)	2(2)	1(1)
Immuno regulatory						1(1)	
Lividin			1(1)	1(1)			
Midkine	1(1)	1(1)	1(1)	1(1)	1(1)	1(1)	1(1)
Nigrocin	1(1)	1(1)	1(1)	1(1)			1(1)
Odorranain			1(1)			8(6)	1(1)
Palustrin			1(1)	1(1)		2(2)	1(1)
Polypedatein				2(1)			
Pelophylaxin	1(1)	1(1)	1(1)	1(1)		1(1)	1(1)
Pancreatic	2(2)	1(1)		1(1)			1(1)
Pleurain							1(1)
Peptide YY	1(1)		1(1)	2(1)		1(1)	1(1)
Serine protease inhibitor						1(1)	1(1)
Uncharacterized			1(1)	1(1)		1(1)	1(1)
All	11(10)	4(4)	15(12)	24(14)	8(6)	28(23)	18(18)

The category of each AMP is shown in parentheses.

**Table 5:** Seven OGs under strong positive selection in the seven anuran species identified in *Site Models* of PAML 4.7.

OGs ID	*M. sangzhiensis*	*O. margaretae*	*P. megacephalus*	*P. nigromaculatus*	*R. dennysi*	*R. omeimontis*	*L. boringii*	Corrected q value	NR annotation
OG02954	MsS|comp35721_c0	OmS|comp91984_c0	PmS|comp62038_c0	PnS|comp51049_c0	RdS|comp53254_c0	RoS|comp205006_c0	VbS|comp39811_c0	0	eIF3C
OG04000	MsS|comp24234_c0	OmS|comp60913_c0	PmS|comp57210_c0	PnS|comp40616_c0	RdS|comp32925_c0	RoS|comp199691_c0	VbS|comp32105_c0	0.003	CAPZB
OG04340	MsS|comp13846_c0	OmS|comp76568_c0	PmS|comp14060_c0	PnS|comp46670_c2	RdS|comp57643_c0	RoS|comp153345_c0	VbS|comp35227_c0	0.007	CD9
OG05658	MsS|comp29431_c0	OmS|comp41010_c0	PmS|comp47485_c0	PnS|comp45618_c0	RdS|comp38617_c0	RoS|comp153557_c1	VbS|comp33211_c0	0.042	ANXA1
OG05703	MsS|comp36086_c0	OmS|comp91549_c0	PmS|comp54574_c0	PnS|comp40281_c0	RdS|comp45282_c0	RoS|comp202769_c0	VbS|comp35512_c0	0.005	TCEA
OG06215	MsS|comp13717_c0	OmS|comp41023_c0	PmS|comp35291_c0	PnS|comp39934_c0	RdS|comp33168_c0	RoS|comp190201_c0	VbS|comp20632_c0	0.008	ATPIF 1
OG06474	MsS|comp17232_c0	OmS|comp105337_c0	PmS|comp60614_c5	PnS|comp21297_c0	RdS|comp57304_c0	RoS|comp153994_c0	VbS|comp41648_c0	0.005	Putative RPL26L1

Note: TCEA, transcription elongation factor A (SII); ATPIF1, ATPase inhibitory factor 1; EIF3C, eukaryotic translation initiation factor 3 subunit C; ANXA1, annexin A1; CAPZB, F-actin capping protein subunit beta 2; CD9, CD9 antigen; and putative RPL26 L1, putative ribosomal protein l26-like 1.
