# Supplementary material for: Comparative transcriptome analyses of seven anurans reveal functions and adaptations of amphibian skin
Source: Sci Rep. 2016 Apr 4;6:24069. doi: 10.1038/srep24069 (PMC4819189; doi:10.1038/srep24069)

Comparative transcriptome analyses of seven anurans reveal functions and adaptations of amphibian skin

Li Huang[#], Jun Li[#], Housseni Anboukaria, Zhenhua Luo, Mian Zhao, Hua Wu[*]

Supplementary Figure S1. The top enriched PI3K-Akt signaling pathway (ko: 04151).

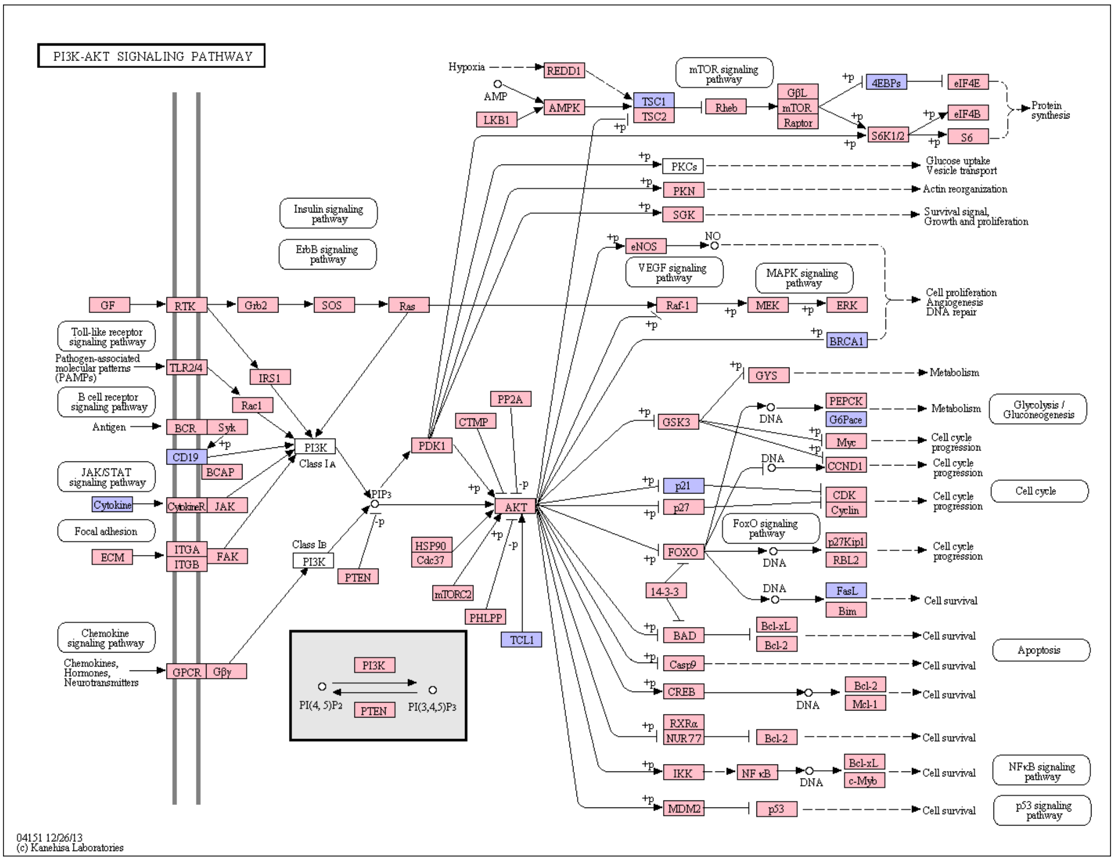

Supplementary Figure S2. Phylogenetic relationship among the seven anurans species. Species names were abbreviated as: *L.boringii* - Lb, *M. sangzhiensis* - Ms, *O. margaretae* - Om, *P. megacephalus* - Pm, *P. nigromaculatus* - Pn, *R. dennysi* - Rd, *R. omeimontis* - Ro, *Xenopus tropicalis* – Xt and *Latimeria chalumnae* – Lc. Xt and Lc were selected to be outgroup species.

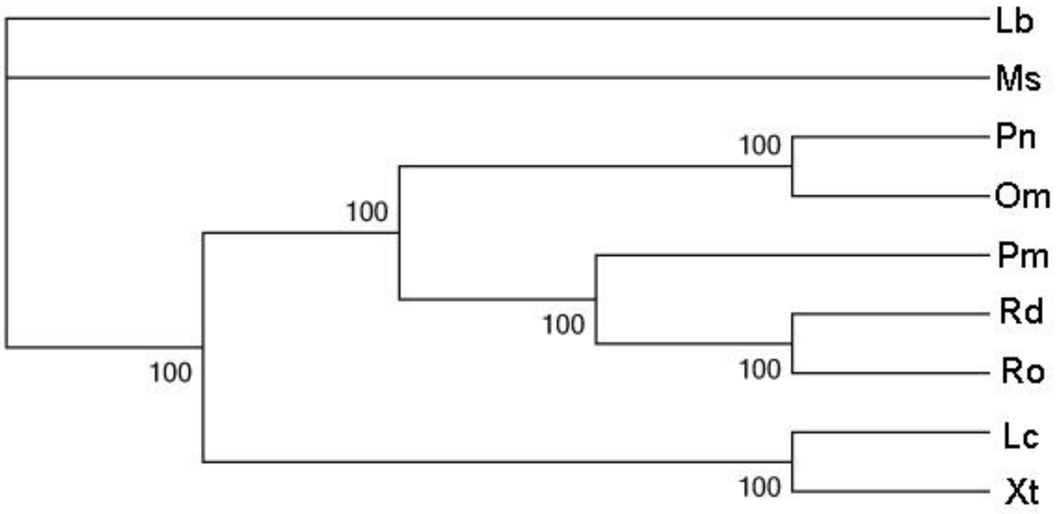

Supplementary Figure S3. The Hematopoletic cell lineage pathway (ko04640) involved by CD9 antigen.

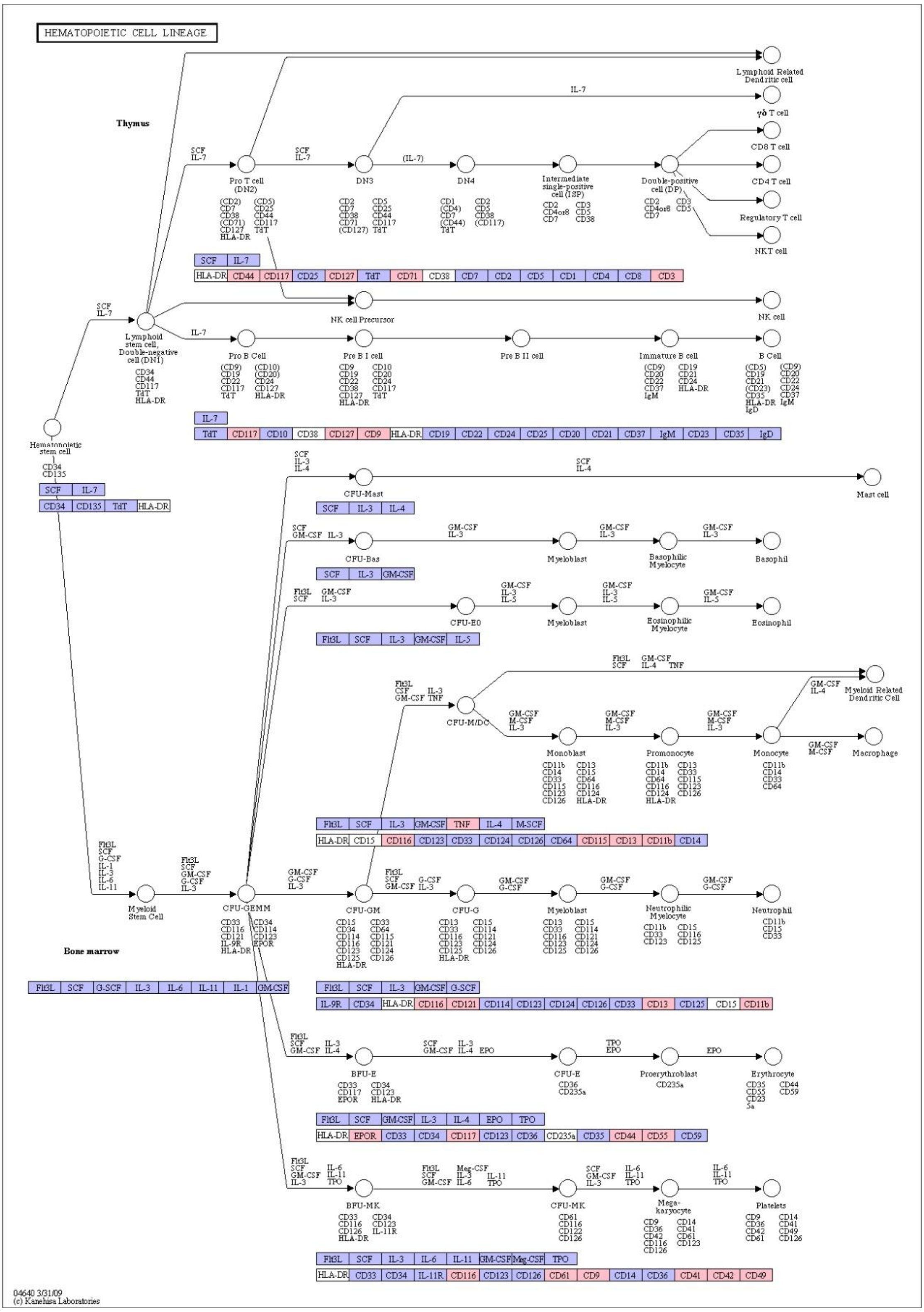

Supplement: Supplementary Information [file srep24069-s1.pdf]
